# Mortality patterns in Vietnam, 2006: Findings from a national verbal autopsy survey

**DOI:** 10.1186/1756-0500-3-78

**Published:** 2010-03-18

**Authors:** Anh D Ngo, Chalapati Rao, Nguyen Phuong Hoa, Timothy Adair, Nguyen Thi Kim Chuc

**Affiliations:** 1 Vietnam Evidence for Health Policy Project, School of Population Health, University of Queensland, 138 Giang Vo Str, Hanoi, Vietnam; 2 Hanoi Medical University, Ton That Tung Str, Hanoi, Vietnam

## Abstract

**Background:**

Accurate nationally representative statistics on total and cause-specific mortality in Vietnam are lacking due to incomplete capture in government reporting systems. This paper presents total and cause-specific mortality results from a national verbal autopsy survey conducted first time in Vietnam in conjunction with the annual population change survey and discusses methodological and logistical challenges associated with the implementation of a nation-wide assessment of mortality based on surveys.

Verbal autopsy interviews, using the WHO standard questionnaire, were conducted with close relatives of the 6798 deaths identified in the 2007 population change survey in Vietnam. Data collectors were health staff recruited from the commune health station who undertook 3-day intensive training on VA interview. The Preston-Coale method assessed the level of completeness of mortality reporting from the population change survey. The number of deaths in each age-sex grouping is inflated according to the estimate of completeness to produce an *adjusted *number of deaths. Underlying causes of death were aggregated to the International Classification of Diseases Mortality Tabulation List 1. Leading causes of death were tabulated by sex for three broad age groups: 0-14 years; 15-59 years; and 60 years and above.

**Findings:**

Completeness of mortality reporting was 69% for males and 54% for females with substantial regional variation. The use of VA has resulted in 10% of deaths being classified to ill-defined among males, and 15% among females. More ill-defined deaths were reported among the 60 year or above age group. Incomplete death reporting, wide geographical dispersal of deaths, extensive travel between households, and substantial variation in local responses to VA interviews challenged the implementation of a national mortality and cause of death assessment based on surveys.

**Conclusions:**

Verbal autopsy can be a viable tool to identify cause of death in Vietnam. However logistical challenges limit its use in conjunction with the national sample survey. Sentinel population clusters for mortality surveillance should be tested to develop an effective and sustainable option for routine mortality and cause of death data collection in Vietnam.

## Background

Vietnam has experienced a demographic transition characterized by decreasing fertility rates in the past two decades and marked improvements in child survival [1,2]. Historical demographic trends have shown that such changes lead to increasing longevity and population aging, accompanied by a shift in disease burden from infectious to non-communicable diseases [3]. Reliable data on patterns of mortality and causes of death is critical to monitor such transitions, and to support the development of evidence-based health policy to minimise avoidable adult mortality. Unfortunately, accurate nationally representative statistics on total and cause-specific mortality in Vietnam are lacking due to inadequate routine information systems [4]. The only available information on mortality levels and patterns is from the Fila Bavi, a demographic surveillance site covering a population of roughly 50,000 located in a rural district about 100 km north of Hanoi [5,6]. These data are limited to a small sample size, narrow geographic coverage, and pertain to a population that does not represent the socio-economic characteristics of Vietnam in general.

To monitor population and demographic indicators in Vietnam, the General Statistics Office (GSO) has been operating an annual population change survey (PCS) in a nationally representative population sample since 2000 [2]. Analysis of 2004, 2005, and 2006 mortality data from this survey indicated severe deficiency in death reporting, ranging between 39% to 59% for the whole country [7]. During 2007 - 2008, a research project was undertaken to improve the completeness of death reporting from the PCS conducted in 2007, and to use verbal autopsy (VA) methods to ascertain the cause of reported deaths. This project is a step towards the expanded use of VA to develop a long-term, national mortality data collection system in Vietnam. This paper presents total and cause-specific mortality results from the project, highlights methodological and logistical challenges in conducting a nation-wide assessment of mortality based on surveys, and discusses future strategies for routine mortality data collection in Vietnam.

## Data and Methods

The annual PCS is implemented in a nationally representative population sample derived through multi-stage stratified cluster sampling. The primary sampling unit is the Enumeration Area (EA) defined in the 1999 population census, which on average comprises approximately 100 households. Each year, a fresh sample of EAs is drawn from these pre-defined EAs. A total of 3840 EAs in the 64 provinces of Vietnam were included in the 2007 survey, covering about 2% of the national population.

The PCS in April 2007 collected information on deaths reported by households to have occurred between 1^st ^April 2006 and 31^st ^March, 2007. For each death, demographic information of the deceased (e.g., age, sex, date of death) and household address were used to locate the household for subsequent VA interviews with the principal care taker of the deceased. For national application of VA, the WHO standard VA questionnaire [8] was adapted to the Vietnamese context, based on experience with VA in the Fila Bavi demographic surveillance site [5,6]. (see Additional file 1).

**Figure 1:**
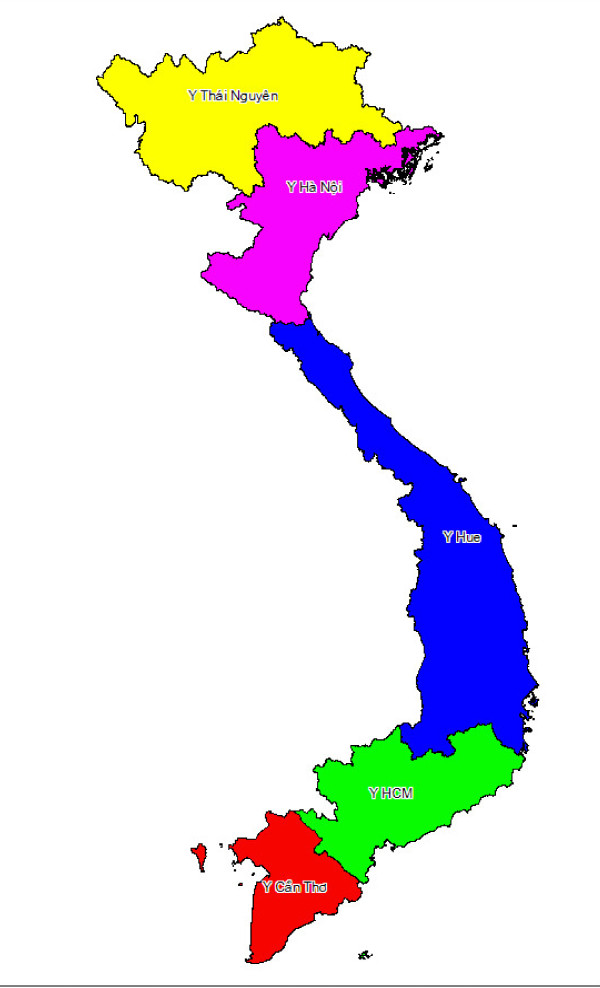
VA interview cluster and the corresponding medical university

Support for training, fieldwork supervision and data management, was provided by 5 medical universities, including Thai Nguyen, Hanoi in the North, Hue in the centre, and Ho Chi Minh and Can Tho in the South. For logistical convenience, 64 provinces were divided into 5 clusters (Figure 1), corresponding with the location of each medical university. VA interviews were completed in 6 months from September 2007 to April 2008, excluding 2 months of New Year (January and February 2008). Filled questionnaires were reviewed by a team of experienced medical doctors at each medical university, who then assigned the causes of death following the standard death certification form [8]. Underlying causes of death were selected and coded using ICD Version10 (ICD-10).

### Statistical analysis

The Preston-Coale method [9] was used to assess the level of completeness of mortality reporting from the GSO PCS (see Additional file 2 for details). The results were used to adjust observed age-specific mortality rates and compute life tables for all Vietnam and each region. Life expectancy estimates at the regional level were calculated along with 95% confidence intervals using the Chiang Silcock method [10].

For statistical presentation, underlying causes of death were aggregated to the International Classification of Diseases (ICD) Mortality Tabulation List 1. Leading causes of death were tabulated by sex for three broad age groups: 0-14 years; 15-59 years; and 60 years and above.

## Results

**Figure 2:**
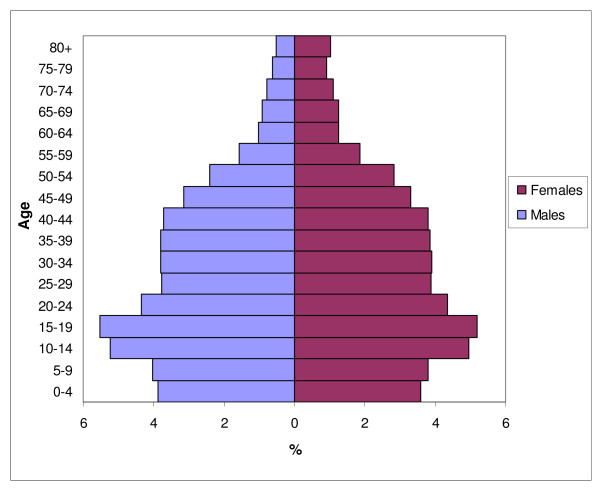
**Population pyramid for Vietnam as observed from PCS 2007**.

Figure 2 shows the age-sex structure of the 2007 survey population. The decline in fertility in the past two decades can be observed from the population proportions in the younger age groups, and this is observed in the population pyramids across all regions.

### All cause mortality

The 2007 PCS identified 7124 deaths for VA follow up interviews. The estimated completeness of mortality reporting in Vietnam was 69% for males and 54% for females. For males, the completeness ranges from 60% to 75%, while for females it ranges from 45% to 62% (Table 1). The figure of over 100% for each sex in Central Highlands is clearly incorrect, and suggests that the assumption of population stability has been breached. Hence, we chose not to compute life tables for this region because of this uncertainty.

**Table 1:** Completeness of mortality reporting and adjusted summary mortality measures by region, males and females, Vietnam, GSO Survey 2007

	Males				Females			
		
Region	**Compl**.	U5MR	Adult	e_0_	**Compl**.	U5MR	Adult	e_0_
Vietnam	69.0	23.0	266	66.4	54.2	26.9	118	73.7
Red River Delta	69.4	17.9	237	67.8	55.2	12.6	127	76.0
Northeast	68.4	39.0	340	60.7	45.7	50.7	134	69.3
Northwest	66.2	51.2	346	60.0	62.3	77.0	114	68.7
North Central	74.6	21.9	302	66.7	58.4	29.9	79	75.5
South Central Coast	64.4	24.4	278	67.3	53.8	26.0	114	74.6
Central Highlands	134.9	-	-	-	119.9	-	-	-
Southeast	73.5	10.0	228	69.1	60.8	10.4	97	76.4
Mekong River Delta	60.2	23.5	259	65.9	45.2	29.9	153	69.9

Compl.: Completeness of mortality reporting (%)

U5MR: Under-five mortality rate per 1,000 births

Adult: Adult mortality rate per 1,000 (15-59 years)

e_0_: Life expectancy at birth (years)

At the national level, estimated life expectancy at birth (LE) is approximately seven years higher for females (73.7 years) compared with males (66.4). These estimates approximate the World Health Organization's estimates for Vietnam in 2006 (75 years for females, and 69 years for males) [11] LE was significantly lower for both males and females in the Northeast and Northwest regions as compared to other regions, based on 95% confidence intervals, These differences largely reflect the variations in the adjusted levels of adult mortality between males and females, and across regions, as listed in Table 1.

### Cause specific mortality

VA interview was completed in 6798 cases (response rate was 95%). The main reasons for non-responses were inability to locate the household due to household moving out, wrong or incomplete address provided by the PCS, or inconsistent use of the name of the head of the household (i.e., nickname, husband's name; first son's name). Only 10 households refused to attend the interview.

Tables 2, 3, 4, 5, 6, 7 present the proportionate cause of death findings for different age groups and all ages. For children aged under 15 years, perinatal conditions is the leading cause of death for both males and females. External causes are prominent in this age group; accidental drowning is a major cause for borth sexes (13% for males, 11% for females) while transport accidents is the 4^th ^leading cause for males (6%).

**Table 2:** Leading causes of death, 0-14 years, Vietnam, 2006-07

Males				Females			
	**Cause**	**N**	**%**		**Cause**	**N**	**%**

1	Perinatal conditions	62	20.9	1	Perinatal conditions	47	22.5
2	Accidental drowning	38	12.8	2	Pneumonia	34	16.3
3	Pneumonia	34	11.4	3	Accidental drowning	23	11.0
4	Transport accidents	19	6.4	4	Congenital malformations	14	6.7
5	Congenital malformations	16	5.4	5	Remainder of diseases of digestive system	13	6.2
	Ill-defined	19	6.4		Ill-defined	16	7.7
	All other causes	109	36.7		All other causes	62	29.7
	Total	297			Total	209	

**Table 3:** Leading causes of death, 15-59 years, Vietnam, 2006-07

Males				Females			
	**Cause**	**N**	**%**		**Cause**	**N**	**%**

1	Transport accidents	264	15.3	1	Transport accidents	60	10.5
2	Cerebrovascular diseases	132	7.7	2	Cerebrovascular diseases	47	8.2
3	HIV/AIDS	105	6.1	3	Liver cancer	31	5.4
4	Liver cancer	97	5.6	4	Suicide	24	4.2
5	Other external causes	86	5.0	5	Other heart diseases	22	3.8
6	Liver diseases	83	4.8	6	Stomach cancer	19	3.3
7	Accidental drowning	65	3.8	7	Colon, rectum, anus cancer	18	3.1
8	Tuberculosis	62	3.6	8	Other cancer of uterus	17	3.0
9	Suicide	61	3.5	9	Accidental drowning	17	3.0
10	Lung cancer	58	3.4	10	Breast cancer	17	3.0
	Ill-defined	124	7.2		Ill-defined	27	4.7
	All other causes	588	34.1		All other causes	273	47.7
	Total	1725			Total	572	

**Table 4:** Leading causes of death, 60 years and above, Vietnam, 2006-07

Males				Females			
	**Cause**	**N**	**%**		**Cause**	**N**	**%**

1	Cerebrovascular diseases	493	24.0	1	Cerebrovascular diseases	467	24.0
2	Chronic lower respiratory diseases	171	8.3	2	Hypertensive diseases	128	6.6
3	Hypertensive diseases	127	6.2	3	Chronic lower respiratory diseases	112	5.8
4	Lung cancer	108	5.3	4	Ischaemic heart diseases	61	3.1
5	Liver cancer	81	3.9	5	Pneumonia	58	3.0
6	Tuberculosis	77	3.7	6	Remainder of diseases of digestive system	52	2.7
7	Ischaemic heart diseases	75	3.6	7	Diabetes mellitus	51	2.6
8	Other heart diseases	57	2.8	8	Tuberculosis	51	2.6
9	Stomach cancer	51	2.5	9	Other heart diseases	47	2.4
10	Liver diseases	50	2.4	10	Remainder of diseases of nervous system	46	2.4
	Ill-defined	239	11.6		Ill-defined	369	19.0
	All other causes	527	25.6		All other causes	503	25.9
	Total	2056			Total	1945	

**Table 5:** Leading causes of death, all ages, Vietnam, 2006-07

Males				Females			
	**Cause**	**N**	**%**		**Cause**	**N**	**%**

1	Cerebrovascular diseases	628	15.4	1	Cerebrovascular diseases	517	19.0
2	Transport accidents	328	8.0	2	Hypertensive diseases	140	5.1
3	Chronic lower respiratory diseases	209	5.1	3	Chronic lower respiratory diseases	127	4.7
4	Liver cancer	179	4.4	4	Pneumonia	102	3.7
5	Lung cancer	166	4.1	5	Transport accidents	86	3.2
6	Hypertensive diseases	156	3.8	6	Liver cancer	75	2.8
7	Tuberculosis	141	3.5	7	Ischaemic heart diseases	74	2.7
8	Diseases of the liver	136	3.3	8	Other heart diseases	72	2.6
9	Ischaemic heart diseases	129	3.2	9	Remainder of diseases of digestive system	71	2.6
10	All other external	125	3.1	10	Remainder of diseases of nervous system	64	2.3
	Ill-defined	382	9.4		Ill-defined	412	15.1
	All other causes	1499	36.8		All other causes	987	36.2
	Total	4078			Total	2727	

**Table 6:** Causes of death by ICD-10 chapter, males, Vietnam, 2007

		0-14 years		15-59 years		60+ years		All ages	
		
ICD-10 Chapter		N	%	N	%	N	%	N	%
I	Infectious and parasitic	22	7.4	195	11.3	90	4.4	306	7.5
II	Neoplasms	11	3.7	355	20.6	406	19.7	775	19.0
IV	Endocrine, nutritional and metabolic	5	1.7	15	0.9	5	0.2	25	0.6
VI	Nervous system	13	4.4	29	1.7	25	1.2	67	1.6
IX	Circulatory system	7	2.4	229	13.3	754	36.7	990	24.3
X	Respiratory system	43	14.5	57	3.3	231	11.2	331	8.1
XI	Digestive system	14	4.7	119	6.9	98	4.8	231	5.7
XIV	Genitourinary	3	1.0	16	0.9	19	0.9	38	0.9
XVI	Perinatal	68	22.9	0	0.0	0	0.0	62	1.5
XVII	Congenital malformations	16	5.4	2	0.1	0	0.0	18	0.4
XVIII	Symptoms etc not elsewhere classified	19	6.4	124	7.2	239	11.6	382	9.4
XIX/XX	Injuries and external causes	72	24.2	544	31.5	128	6.2	744	18.2
	All other chapters	4	1.3	40	2.3	61	3.0	109	2.7
	Total	297		1725		2056		4078	

**Table 7:** Causes of death by ICD-10 chapter, females, Vietnam, 2007

		0-14 years		15-59 years		60+ years		All ages	
		
ICD-10 Chapter		N	%	N	%	N	%	N	%
I	Infectious and parasitic	16	7.7	38	6.6	85	4.4	139	5.1
II	Neoplasms	5	2.4	168	29.4	256	13.2	429	15.7
IV	Endocrine, nutritional and metabolic	3	1.4	14	2.4	54	2.8	71	2.6
VI	Nervous system	10	4.8	15	2.6	66	3.4	91	3.3
IX	Circulatory system	7	3.3	101	17.7	706	36.3	814	29.8
X	Respiratory system	37	17.7	23	4.0	187	9.6	247	9.1
XI	Digestive system	14	6.7	21	3.7	81	4.2	116	4.3
XIV	Genitourinary	0	0.0	11	1.9	21	1.1	32	1.2
XV	Pregnancy & childbirth	3	1.4	5	0.9	1	0.1	9	0.3
XVI	Perinatal	47	22.5	0	0.0	0	0.0	47	1.7
XVII	Congenital malformations	14	6.7	0	0.0	0	0.0	14	0.5
XVIII	Symptoms etc not elsewhere classified	16	7.7	27	4.7	369	19.0	412	15.1
XIX/XX	Injuries and external causes	37	17.7	126	22.0	82	4.2	245	9.0
	All other chapters	0	0.0	23	4.0	38	2.0	61	2.2
	Total	209		572		1946		2727	

The cause-specific mortality results for the 15-59 years age group provides insight into the reasons for the large sex differential in the adult mortality rate. The leading causes of male mortality are transport accidents, cerebrovascular diseases HIV/AIDS, liver cancer and other diseases of liver. Tuberculosis is also a leading cause for this age group (4%). For females, there are some similarities in the rank order of leading causes, but with clearly fewer deaths from each cause.

In both sexes, nearly one fourth of deaths above 60 years are caused by cerebrovascular diseases. Other major causes include chronic lower respiratory diseases, hypertensive diseases, ischaemic heart diseases and tuberculosis. It is interesting to note that lung cancer is a major cause in men, probably a result of the tobacco epidemic. Ill-defined causes are also prominent in this age group, underscoring the limitations of VA in cause of death ascertainment in the elderly. The leading causes of death across all ages (see Table 5) indicate that non-communicable diseases and injuries account for the bulk of the burden from premature mortality in Vietnam.

In addition to leading causes of death, a comprehensive summary of the cause of death distribution by broad age group and sex is presented in Tables 6 and 7. For ease in interpretation, causes of death were aggregated to ICD 10 chapters for categorical presentation. These tables enhance the appreciation of the magnitude of broad categories such as injuries, circulatory system diseases, infectious diseases etc as causes of mortality at different ages.

## Discussion

This paper presents the first ever findings on nation-wide total and cause specific mortality patterns in Vietnam. Although there are several constraints in the quality of the data, the broad findings from the study have important implications for Vietnam. Firstly, the adjusted estimates of life expectancy at birth and levels of adult mortality are consistent with previously observed mortality time trends in Vietnam [12] and demonstrate steady improvements in population health status in Vietnam over the past three decades. Secondly, important differences were noted when our results on cause-specific mortality are compared with the findings from the VA study of 189 deaths in Fila-Bavi in 1999 [5]. While diseases of the circulatory system, cancers, and accidents were the three prominent causes of deaths among all age groups in both studies, infectious diseases, prenatal and neonatal causes were not among 10 leading causes in our study. These differences are important, but unsurprising, given the relatively small sample and rural setting of the Fila-Bavi demographic surveillance site.

Overall, the use of VA has resulted in about 10% of deaths being classified to ill-defined conditions among males, and 15% in females, which is acceptable given the challenges in diagnosing causes of death from this method. In old age (60 years or older), the proportion of deaths classifiable is lowest compared to that in other age groups, a similar finding to studies in India that reported VA is a less reliable to ascertain cause of death for older age [13,14].

Despite the incompleteness of death recording, the proportionate mortality by cause at different ages provides an empirical basis for understanding health priorities. The findings suggest a clear need to improve health services to control perinatal mortality, and the need for evidence based interventions to reduce deaths from traffic accidents and drowning. Among adults, the observation that cerebrovascular disease causes about 5 times the number of deaths as ischemic heart diseases (IHD)calls for more detailed research into the epidemiology of these conditions, and the implementation of primary and secondary prevention strategies. This ratio could be skewed on account of IHD deaths being misclassified as deaths from hypertensive diseases, which is observed to cause about double the proportion of deaths from IHD at ages 60 and over. This requires careful evaluation through assessment of the reliability and/or validity of VA application in Vietnam. Additional findings for males include the emergence of HIV/AIDS as a leading cause of death at ages 15-59 years, and tuberculosis as the seventh leading cause of death at all ages. All these findings signify the need to improve surveillance and treatment programs for these conditions.

From an operational perspective, integration of activities to measure cause-specific mortality with the existing annual national PCS offered several advantages. Firstly, it saved resources that otherwise would be needed for identification of deaths in a nationally representative sample, given incomplete vital registration in Vietnam. Secondly, mortality data provided by GSO is legally recognized by the government of Vietnam, and therefore, the results from the study can inform policy development. In this regard, activities in this project to strengthen death recording by GSO staff did result in improvement in the completeness of data. More importantly, however, the activity enabled 5 medical universities throughout the country to develop experience in the systematic investigation of cause of death on a nation-wide scale, using the VA method. This has created an institutional network in different regions, which is a sound platform for routine and sustained implementation of cause-specific mortality data collection systems in all parts of Vietnam.

However, despite the operational advantages described above, the GSO annual PCS presented significant challenges in translating the data collected into reliable information for routine mortality measurement. Firstly, a principal issue with the data is the low completeness of deaths recorded by surveyors (see Additional File 2). Secondly, there were several challenges in the implementation of VA interviews in conjunction with the survey (see Additional File 1). Hence, while this exercise has proved to be a valuable experience as the first ever national level mortality and cause of death data collection in Vietnam, more effective and sustainable options are required for routine implementation. To meet this challenge, sentinel mortality surveillance sites are being tested in 2009. The proposed sentinel sites comprise 192 communes in 16 provinces, covering an optimal sample population given the mortality profile for Vietnam [15]. At the commune level, data collection efforts involve the collaboration of the official civil registration system, the commune health station, and local 'population collaborators' from the Population and Family Planning Department of the Ministry of Health. The tools, methods, and experiences from the current study would be used together with technical support from the five medical universities. Continuous data collection in these sites over the next 5-10 years, supported by appropriately designed studies to validate reported causes of death, would yield valuable evidence on current mortality patterns and trends, to inform health policy and epidemiological research in Vietnam.

## Conclusion

This study provides evidence that verbal autopsy can be a viable tool to identify cause of death in Vietnam. However, its use in conjunction with the national sample survey is limited by incomplete death reporting as well as logistical challenges in implementing household VA interviews. The proposed sentinel mortality surveillance sites being tested in 2009, using tools, methods and experiences from the current study, will help better understand the applicability of the VA method and develop effective and sustainable systems for routine mortality assessment in Vietnam.

## List of abbreviations used

EA: Enumeration area; GSO: General Statistics Office; Ischemic heart diseases: IHD; LE: Life expectancy at birth; PCS: Population change survey; VA: Verbal autopsy.

## Competing interests

The authors declare that they have no competing interests.

## Authors' contributions

CR conceived research ideas. AN, CR, NTPH and NTKC developed research protocol, data collection tools, and conduct data collection and data entry. TA performed data analysis. AN drafted the manuscript with input from all other authors. All authors reviewed and approved the final version.

## Supplementary Material

Additional file 1 **Verbal autopsy methods and limitations**. This file describes the application of the VA method in the study, the challenges and limitations of VA implementation. Click here for file

Additional file 2 **Completeness of death reporting in the GSO survey, 2007**. This file presents measures undertaken to improve the completeness of death reporting in the 2007 GSO survey and describes methods used to estimate the completeness of death recording in this survey. Click here for file
