# Supplementary material for: Mortality patterns in Vietnam, 2006: Findings from a national verbal autopsy survey
Source: BMC Res Notes. 2010 Mar 18;3:78. doi: 10.1186/1756-0500-3-78 (PMC2851717; doi:10.1186/1756-0500-3-78)
Supplement: Additional file 1 — Verbal autopsy methods and limitations. This file describes the application of the VA method in the study, the challenges and limitations of VA implementation. [file 1756-0500-3-78-S1.DOC]

**Verbal autopsy methods and limitations**

For national application of VA, the WHO standard VA questionnaire was adapted to the Vietnamese context, based on experience with VA in the Fila Bavi demographic surveillance site.^1^ The questionnaire consists of two modules: one for deaths in neonates and children below 12 years; and the other for adolescents and adults. Basically, the questionnaire captures information on the following: (i) Family background and demographic characteristics; (ii) Signs and symptoms check list; (iii) Open-ended questions to elicit information on the terminal illness leading to death; and (iv) information from contact with health services during illness preceding death. The English version of the questionnaire was translated and back translated to Vietnamese and pilot tested before being used in the project. Findings from the pilot test and from focus group discussions with a sample of interviewers and respondents suggested specific changes to the questionnaire through the use of local terms to indicate individual symptoms. These changes were intended to maximize the comprehension of and desensitize the respondents to specific questions. In the final version of the questionnaire, the number of questions and response items were essentially maintained as in the original English version, although there were some changes to the order of questions to ensure smooth flow of the interview.

The VA interviewers chosen for this project were nurses, or midwifes recruited from communal health stations or district health centres. Data collectors underwent 3-day intensive training on VA interviewing skills. The training emphasized desensitizing techniques and communication skills to motivate the principal care taker of the deceased to participate in the interview and give appropriate answers. Furthermore, training covered specific content of the questionnaire, the underlying intent of each question, and specific instructions for coding responses. Also, the training addressed the issues of under-reporting, recall bias, and other pertinent problems and limitations in the GSO’s annual survey. The training methods included lecture presentation, group discussions, role-play, and hands-on practice. The potential bias arising from prior medical knowledge of the data collectors was also addressed, with suitable instructions to ask and record each question as per the protocol.

VA data collection was administered through 5 medical universities, including Thai Nguyen, Hanoi in the North, Hue in the centre, and Ho Chi Minh and Can Tho in the South. For logistical convenience, 64 provinces were divided into 5 clusters (see Figure 1 in manuscript), corresponding with the location of each medical university. Each participating medical university was responsible for conducting VA data collection, reviewing VA questionnaire to determine the causes of death, and coding the causes of death identified in the respective cluster. VA interviews were completed in 6 months from Sep. 2007 to April 2008, excluding 2 months of New Year (January and February, 2008).

Filled questionnaires were reviewed by a team of experienced medical doctors at each medical university, who then assigned the causes of death following the standard death certification form.^1^ Underlying causes of death were selected and coded using ICD Version10 (ICD-10), by medical doctors who were trained in ICD coding and had good command of English, being able to use the English version of the ICD-10 coding manual.

**Challenges and limitations of VA implementation**

There were several challenges in the implementation of VA interviews, some of which also influenced the utility of the collected data in ascertaining causes of death. From a logistics perspective, the data collection was hampered by wide geographic dispersal of deaths, owing to the nature of the small size of population clusters (EA) across districts and provinces. This was compounded by the need for multiple household visits to complete the VA interview in some cases, due to non-availability of the appropriate respondent.

In terms of actual data collection, variations in the characteristics of both respondents and interviewers could have influenced data quality. In particular, the quality of responses to structured questions required adequate comprehension by respondents, and the quality of recorded information in the open ended section of the questionnaire is dependent on strong interviewing skills.^2^ In a number of cases, the responses to the symptom checklist did not appear to be helpful for diagnosis, due to a high numbers of missing items or ‘don’t know, don’t remember’ responses. Under such circumstances, reviewing physicians assigned a probable cause of death primarily relying on respondents’ narratives in an open-ended question of the terminal illness that leads to death. Also, the long period (up to 2 years) between death and the VA interview could have introduced recall bias. Finally, respondents were sometimes hesitant in revealing details about causes of death that were considered sensitive such as deaths due to HIV/AIDS, drug-related deaths, or infant deaths. These aspects require attention during future implementation of VA for population health assessment in Vietnam.

**References**

1. WHO, **Verbal autopsy standards. Ascertaining and Attributing Cause of Death**. 2007: Geneva.
2. Lulu K, Berhane, Y. **The use of simplified verbal autopsy in identifying causes of adult death in a prominantly rural population in Ethitopia***.* BMC Public Health, 2005. 5(58).
