# Supplementary material for: Mortality patterns in Vietnam, 2006: Findings from a national verbal autopsy survey
Source: BMC Res Notes. 2010 Mar 18;3:78. doi: 10.1186/1756-0500-3-78 (PMC2851717; doi:10.1186/1756-0500-3-78)
Supplement: Additional file 2 — Completeness of death reporting in the GSO survey, 2007. This file presents measures undertaken to improve the completeness of death reporting in the 2007 GSO survey and describes methods used to estimate the completeness of death recording in this survey. [file 1756-0500-3-78-S2.DOC]

**Completeness of death reporting in the GSO survey, 2007**

The completeness of death recording during household mortality surveys is known to be limited on account of recall bias, which could either be due to misinterpretation of the reference period for data collection, or genuine omission.^1^ Analysis of data collected during the annual PCS conducted by the General Statistics Office, Vietnam revealed significant under-reporting of deaths over several years.^2^ In view of this situation, specific measures were undertaken to improve the completeness of data collection in the 2007 survey.

A series of focus group discussions were conducted with GSO data collectors during the two months preceding the survey, to identify problems with death reporting in the PCS. The findings were used to develop supplementary training modules for GSO field surveyors. The initial session of training focussed on dissemination and discussion of results from previous surveys to field staff, in order to clearly highlight the deficiencies in the PCS mortality data. The training then focused on desensitizing interviewers’ perceptions about death enquiry, and strengthening interviewing techniques and recording of responses, to improve the utility of mortality data from the 2007 survey. Overall, these measures resulted in a 20% increase in the numbers of deaths reported in previous surveys, which could be attributed to the special efforts undertaken to improve the quality of data collection through focus group discussions and supplementary training.

Despite these measures and the observed improvements, demographic analyses shown in Table 1 of the manuscript identified that underreporting of deaths is still a problem, and the overall completeness remained only 60-75% across the regions. Discussions with surveyors and supervisors identified that there are communication barriers in asking mortality questions, time constraints in data collection, and respondent fatigue in answering lengthy questionnaires on an annual basis, and all these factors result in under-reporting of deaths.

**Estimating completeness of death recording**

The Preston-Coale method was used to estimate completeness of death recording.^3^ This method estimates completeness based on basic life table inputs (i.e., age distribution of deaths and population), as well as an estimate of the population growth rate. The Preston-Coale method assumes a stable population, that is, where fertility and mortality have been constant in recent decades, there has been no migration, and hence there is a constant rate of population growth. The Preston-Coale method computes the average level of completeness of age groups five years and above. For the purposes of this study, the level of completeness for each sex is used to inflate the number of deaths across all ages in the life table. The population growth rate used in the Preston-Coale method computation is the inter-censal growth rate from 1989 to 1999, the years of the two most recent population censuses. The annual growth rate for this period was 1.82% for males and 1.59% for females.

We applied the adjustment factor for completeness of registration to the under-five age group as well, although the use of indirect methods for such adjustments not recommended at these ages ^4^. However, our adjustments yielded under-five mortality risks (23, 27, and 25 per 1000 for males, females, and both sexes respectively) that closely approximate such estimates from UNICEF for Vietnam in 2006 (28 per 1000 for both sexes).^5^

**References**

1. Preston SH. **Mortality data collection in the context of data needs. Data bases for mortality measurement**. Proceedings of the United Nations / World Health Organization Working Group on Data Bases for Measurement of Levels, Trends, and Differentials in Mortality. 1981, Bangkok.

2. General Statistics Office, **Assessment of completeness of mortality data in 2004, 2005, and 2006 population change survey**, 2007: Hanoi. Unpublished

3. Preston S, Coale AJ, Trussell J, and Weinstein M. **Estimating the completeness of reporting of adult deaths in populations that are approximately stable***.* Popul Index, 1980. 46(2): 179-202.

4. Preston SH. **Use of Direct and Indirect techniques for estimating the completeness of registration systems. Data bases for mortality measurement**. Proceedings of the United Nations / World Health Organization Working Group on Data Bases for Measurement of Levels, Trends, and Differentials in Mortality. 1981, Bangkok.

5. UNICEF. **The state of the World's children, 2008. Table1. Basic Indicators.**

[cited 2010 February]; Available from: http://www.unicef.org/sowc08/docs/sowc08.pdf
